# FAIM2, as a novel diagnostic maker and a potential therapeutic target for small-cell lung cancer and atypical carcinoid

**DOI:** 10.1038/srep34022

**Published:** 2016-09-28

**Authors:** Hio Chung Kang, Jong In Kim, Hee Kyung Chang, Gavitt Woodard, Young Sik Choi, Ja-Lok Ku, David M. Jablons, Il-Jin Kim

**Affiliations:** 1 Thoracic Oncology Laboratory, Department of Surgery, University of California San Francisco, San Francisco, CA, USA; 2 Comprehensive Cancer Center, University of California San Francisco, San Francisco, CA, USA; 3 Department of Thoracic and Cardiovascular Surgery, Kosin University College of Medicine, Busan, Republic of Korea; 4 Department of Pathology, Kosin University College of Medicine, Busan, Republic of Korea; 5 Department of Internal Medicine, Kosin University College of Medicine, Busan, Republic of Korea; 6 Laboratory of Cell Biology, Cancer Research Institute, Seoul National University College of Medicine, Seoul, Republic of Korea

## Abstract

Lung neuroendocrine (NE) tumors are a heterogeneous group of tumors arising from neuroendocrine cells that includes typical carcinoid, atypical carcinoid, small cell lung cancer (SCLC), and large cell NE cancer. The subtyping of NE tumors is based on the number of mitoses per high powered field and the presences of necrosis. However, the best diagnostic criteria to differentiate various subtypes of lung NE tumors remains controversial and few diagnostic markers distinguish typical and atypical carcinoid. In this study, we show that *FAIM2*, an inhibitory molecule in the Fas-apoptosis pathway, is significantly overexpressed in SCLC compared to non-small cell lung cancer. In addition, *FAIM2* expression is significantly higher in atypical carcinoid than typical carcinoid. As atypical carcinoid has been shown to have worse clinical outcomes than typical carcinoid, our data suggests that *FAIM2* may be a useful diagnostic marker for atypical carcinoid. Knockdown of FAIM2 expression increases Fas-induced apoptotic cell death in SCLC cells. Etoposide treatment combined with FAIM2 inhibition also shows modest but significant reduction of viable SCLC cells. Taken together, our results suggest that FAIM2 is a potential NE tumor marker with higher expression in atypical carcinoid and SCLC, and could be a new therapeutic target for SCLC.

Lung cancer is the second most common cancer worldwide comprising ~12% of all human cancers and is the leading cause of cancer mortality in the United States and worldwide. The majority of lug cancer is non-small cell lung cancer (NSCLC) comprises ~85% of new lung cancer diagnoses^1^,^2^,^3^,^4^. The remainder of new lung cancer diagnoses are small cell lung cancer (SCLC) which accounts for ~15% of new lung cancer diagnoses, and carcinoid tumors which make up the remaining small percentage of primary lung tumors. The neuroendocrine (NE) tumors large cell neuroendocrine carcinoma (LCNEC), SCLC, typical carcinoid and atypical carcinoid all originally arise from neuroendocrine cells but these tumors behave differently and carry very different prognoses^1^,^2^,^3^,^4^.

Lung NE tumors have been classified into the following criteria by WHO in 2004 based on mitotic rate and the presence of necrosis^1^,^2^,^3^. Typical carcinoid is a low-grade NE lung cancer (less than 2 mitoses per 10 high power fields (HPF) and no necrosis) and an atypical carcinoid (2–10 mitoses per HPF and/or no necrosis) is an intermediate-grade NE cancer in the lung^1^,^2^,^3^. High-grade neuroendocrine (NE) lung cancers (more than 10 mitoses per HPF and necrosis) are classified as SCLC or LCNEC^1^,^2^,^3^. The sub-classification of NE tumors has been limited by a lack of diagnostic markers, especially for the high-grade NE tumors SCLC and LCNEC. In order to diagnose these two high-grade tumors additional cytological, radiological, and histological analysis are required^2^. Similarly, it is often challenging to discriminate between the low and intermediate grade NE tumors typical and atypical carcinoids as there is no histological marker specific for each type. Atypical carcinoids grow more quickly and have worse clinical outcomes than typical carcinoid^2^,^5^,^6^, and potential new histological or molecular markers for atypical carcinoid are evaluated based on their correlation to worse clinical outcomes. For example, Ki-67 has previously been correlated with prognosis of NE tumor patients and therefore it has been suggested that Ki-67 could be added to the classic markers of mitosis and necrosis to help classify NE tumors^2^,^7^. However, its role in the classification and diagnosis of lung NE tumors remains controversial.

SCLC has the worst prognosis of all primary lung cancers with a 5-year survival rate of less than 7%^8^. SCLC is strongly associated with smoking and has been shown to have more than 10 times higher mutation rates than prostate cancers by sequencing analysis^3^,^9^. While advances have been made in NSCLC treatment due to targeted therapies and the comprehensive discovery of molecular targets such as *EGFR* and *EML4-ALK*^10^,^11^,^12^, no significant progress has been made in the treatment of SCLC over the last decade. Mutations or loss of *TP53* and *RB1* have been reported in 75–100% of SCLC patients, but there is no FDA approved therapy to target these mutations. Limited access to SCLC tissue for research purposes may be one reason why the development of targeted therapies for SCLC has lagged behind research in NSCLC. It has been suggested that some NSCLC cells with *EGFR* mutations can be converted into SCLC cells after treatment with tyrosine kinase inhibitors (TKIs) or even synchronously without any treatment^13^. Interestingly, all of these NSCLC to SCLC transformed cells harbored *RB1* loss^13^. Adding to the complexity, around 2–14% of SCLC has an NSCLC component^13^,^14^, which makes developing targeted therapy to heterogeneous tumors a challenge.

In SCLC the best survival outcomes are achieved with combined platinum-based chemotherapy (cisplatin and etoposide) with radiation therapy for limited-stage SCLC patients and platinum-based chemotherapy alone in patients with extensive disease^3^. Although most SCLC patients (60–70%) initially respond to treatment, almost all develop resistance to treatment and relapse within 6 months. After resistance to first-line therapy develops the only approved second-line therapy is the topoisomerase inhibitor I topotecan^3^, which generates a response in only 3–25% of patients^3^,^15^. It was reported that Amrubicin, a topoisomerase inhibitor II, was superior to topotecan in Japanese SCLC patients, but this finding has not been observed in Western patient populations^16^. Many additional drugs have been tested in SCLC clinical trials, however none have shown better treatment efficacy.

The lack of treatment options in SCLC highlights the urgent need for understanding mechanisms of SCLC development, drug resistance, and the development of targeted therapies in SCLC. In addition to expanding treatment options, better survival in SCLC could be achieved by diagnosing tumors at an earlier stage. While circulating tumor cells (CTC) have recently been suggested as potential diagnostic markers for SCLC^17^, none are close to approval for clinical use. In order to develop an efficient early diagnostic method for SCLC it is important to identify SCLC–specific or dominant molecular markers. Several markers such as synaptophysin, chromogranin, or CD56 (NCAM) have been used to diagnose SCLC and other NE tumors in the lung^2^. However, more specific histological markers should be developed to improve diagnostic accuracy between the different subtypes of lung NE tumors.

Regulation of apoptosis is important for both tumor cell survival and death and chemotherapy targeted at apoptotic pathways has been broadly approached. Anti-apoptotic inhibitors including anti-Bcl2 agents have been tested without success in SCLC^4^,^18^,^19^. In our search for molecular markers of SCLC from SCLC genomic data we identified *FAIM2* (Fas apoptotic inhibitory molecule 2 or LFG). FAIM2 inhibits Fas-mediated cell death^20^ and is highly overexpressed in SCLC cell lines. It has been suggested that frequent loss of Fas expression in SCLC cells is a mechanism by which SCLC cells avoid Fas-mediated apoptosis^21^. Overexpression of Fas ligand (FasL) followed by the loss of Fas in SCLC may be associated with cytotoxic T cell expressing Fas^21^. However almost nothing is known about the role of *FAIM2* in the development of SCLC or other NE tumors in lung.

In this study, we demonstrate that immunohistochemical detection of FAIM2 expression outperforms the known NE tumor marker synaptophysin in SCLCs and atypical carcinoids. This suggests that *FAIM2* is a new diagnostic marker of SCLC, atypical carcinoid, and other pulmonary NE tumors. In addition, combined treatment with etoposide and FAIM2 inhibition reduced viability of SCLC cells, which suggests that FAIM2 inhibition may represent a new therapeutic option in SCLC.

## Results

### Expression analysis of genes involved in Fas-apoptosis pathway reveals elevated expression of FAIM2, anti-apoptotic gene, in SCLC cells

**Figure 1:**
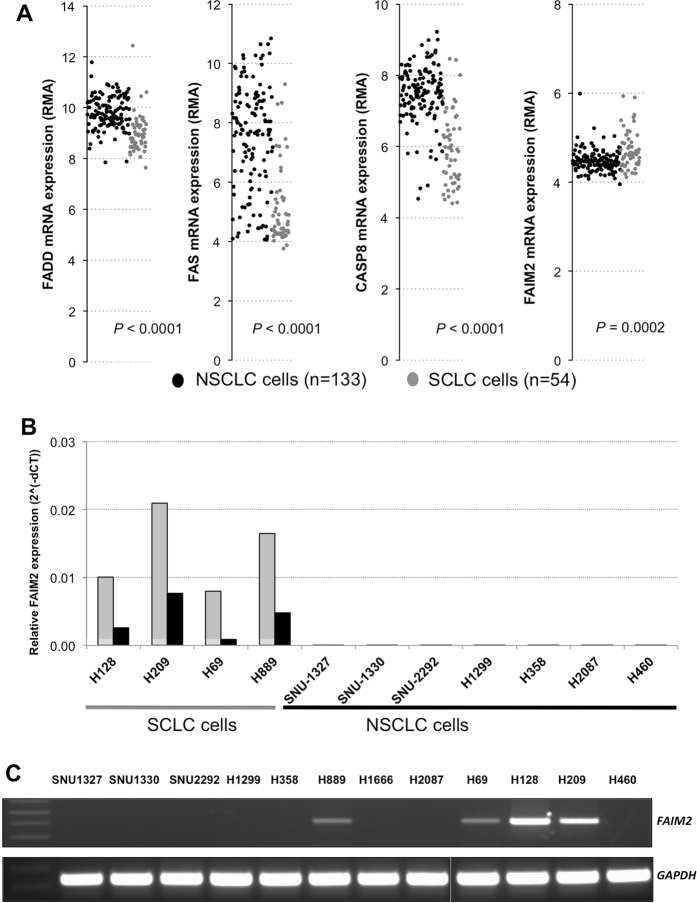
Significantly elevated expression of *FAIM2* in SCLC compared to NSCLC cell lines. (**A**) Microarray data from a total of 187 lung cancer cell lines (133 NSCLC and 54 SCLC) available in the CCLE database were analyzed for Fas-apoptosis pathway genes including *FADD, FAS, CASP8*, and *FAIM2*. While pro-apoptotic genes, *FADD, FAS*, and *CASP8* were significantly underexpressed in SCLC cells compared to NSCLC cells (*p* < 0.0001), *FAIM2* expression was significantly overexpressed in SCLC cells compared to NSCLC cells (*p* = 0.0002). (FADD: 1.8 fold increase of expression in SCLC, FAS: 4.9 fold increase of expression in SCLC, CASP8: 3.0 fold increase of expression in SCLC: FAIM2: 0.8 fold decrease of expression in SCLC- Fold change was calculated from the average values). NSCLC: black dot. SCLC: gray dot. RMA: Robust Multi-array Average. (**B**) Four SCLC and seven NSCLC cell lines were analyzed by real-time quantitative RT-PCR for *FAIM2* mRNA expression. Two Taqman assays detecting exons 2–3 (colored in gray) and exons 5–6 (colored in black) in the *FAIM2* transcript were used for *FAIM2* expression analysis. *FAIM2* expression was significantly higher in SCLC than those of NSCLC cell lines (*p* < 0.0001). (**C**) The result from Taqman assays was confirmed by semi-quantitative RT-PCR. *FAIM2* expression was clearly detected in four SCLC cell lines while no clear band was detected in NSCLC cell lines. GAPDH was used as a housekeeping gene control.

Evading programmed cell death is an important mechanism that tumor cells exploit to resist chemotherapy and survive indefinitely. SCLC is known for its ability to quickly develop chemotherapy resistance mechanisms and SCLC genes involved in apoptosis signaling are an area of interest. Taking advantage of publicly available genomic resources, we explored the Cancer Cell Line Encyclopedia (CCLE) database of genome-wide expression data of 1000 cancer cell lines (http://www.broadinstitute.org/ccle). Expression data of genes involved in Fas-apoptotic pathway, a key apoptotic signaling pathway, were obtained for 187 lung cancer cell lines. These cell lines were categorized into NSCLCs and SCLCs based on the available histology subtype information. By comparing expression level of Fas-apoptosis related genes, we observed that the relative expression of *FAS*, Fas-associated protein with death domain (*FADD*), and caspase 8 (*CASP8*) were significantly suppressed in SCLC cells (Fig. 1A). Lack of procaspase 8 expressions has been previously observed in SCLC cells^22^. Interestingly, *FAIM2*, a Fas apoptosis inhibitory molecule 2, showed significantly elevated expression levels in SCLC cells (*p *= 0.0002) (Fig. 1A). *FAIM2* is known as an anti-apoptosis gene that directly interacts with Fas receptor and inhibits apoptosis by interfering with caspase 8 activation^23^. We further focused our attention on the inverse correlation of *FAIM2* expression with known proapoptotic genes *FAS*, *FADD*, and *CASP8*.

To further validate our observations from the CCLE database, we performed quantitative real-time PCR (qRT-PCR) for *FAIM2* expression in 12 lung cancer cell lines. Two different *FAIM2* probes were used for qRT-PCR validation. As predicted by our CCLE database observations, all 4 SCLC cell lines (H69, H128, H209, and H889) showed highly elevated *FAIM2* expression in two different assays detecting exons 2–3 and exons 5–6 in the *FAIM2* transcript (Fig. 1B) compared to 8 NSCLC cell lines (*p *< 0.0001). This result was confirmed again by semi-qRT-PCR (Fig. 1C). These data suggest that *FAIM2* overexpression may be a potential molecular marker in SCLCs.

### FAIM2 overexpression in neuroendocrine tumor cells and lung carcinoids

**Figure 2:**
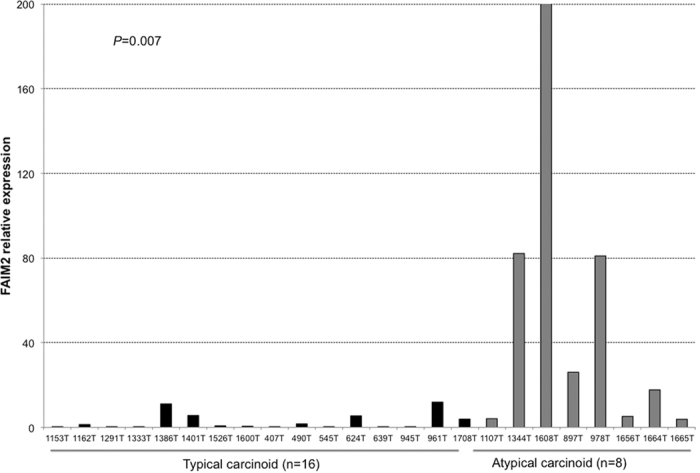
Higher *FAIM2* mRNA expression in atypical carcinoid than in typical carcinoid tumors in the lung. Taqman analysis of *FAIM2* mRNA expression was done in 16 frozen typical carcinoid and 8 atypical carcinoid tumors in the lung. *FAIM2* was significantly more highly expressed in atypical carcinoid tumors while low or no expression of *FAIM2* was detected in typical carcinoid tumor samples (*p* = 0.007).

SCLC is classified as a pulmonary neuroendocrine tumor due to its neuroendocrine origin^2^. To test the hypothesis that *FAIM2* could act as a novel NE tumor marker, we examined the CCLE database for NE tumor cell lines with *FAIM2* expression data, and found 17 neuroblastoma (NB) cell lines. Robust Multi-array Average (RMA) values for *FAIM2* expression were retrieved and compared with those of NSCLC and SCLC cells. As suspected, *FAIM2* expression was significantly more elevated in NB cells than in NSCLC cells (*p *= 0.0015) (Figure S1). In contrast, three proapoptotic genes, *FAS, FADD*, and *CASP8,* showed relatively reduced expression in NB cells, similar to what was observed in SCLC cells (*p *< 0.0001) (Figure S1). To further elucidate *FAIM2* as a novel NE tumor marker, we gathered available pulmonary NE tumor information from our tissue bank at UCSF. Among lung NE tumors, 24 lung snap frozen carcinoid and matched normal tissues were available. We extracted RNA from 24 lung carcinoids (16 typical and 8 atypical) and their matched normal tissues and performed qRT-PCR for *FAIM2* expression. Interestingly, *FAIM2* expression in atypical carcinoids was significantly higher than in typical carcinoids (*p *= 0.007) (Fig. 2). Differentiating atypical from typical carcinoids can be challenging on histopathology but is an important distinction as these two subtypes have different prognostic outcomes^5^. Therefore, the differential expression of *FAIM2* between typical and atypical carcinoid tumors is a useful and meaningful diagnostic marker.

### Immunohistochemistry (IHC) evaluation of FAIM2 in comparison to known NE tumor markers in pulmonary NE tumors

Currently the NE tumor markers chromogranin, synaptophysin, neuron-specific enolase (NES), and CD56 are widely used in immunohistochemistry (IHC) analysis^2^ however none have perfect sensitivity or specificity for SCLC. Identifying more specific SCLC markers that outperform the currently available markers would be very useful in establishing a diagnosis in patients with challenging histopathology.

**Figure 3:**
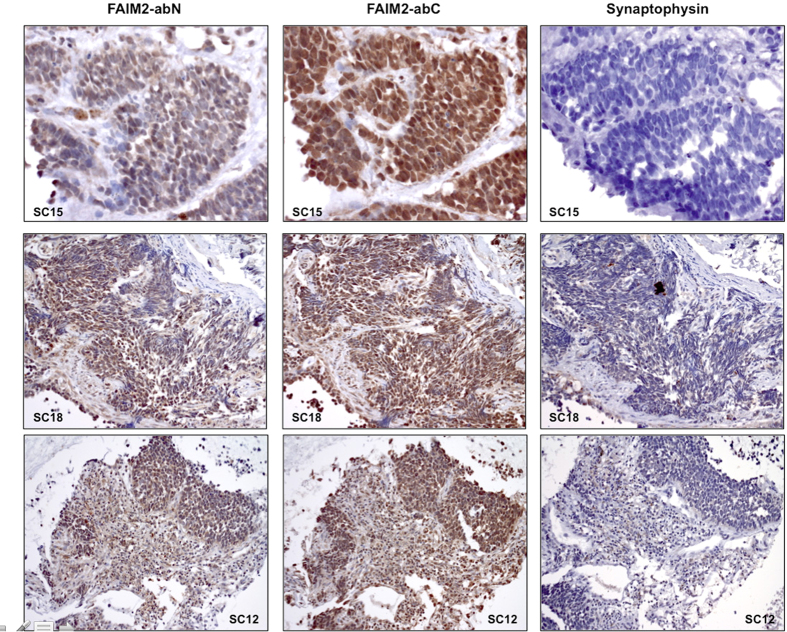
Immunohistochemistry (IHC) of FAIM2 expression in SCLCs. Two different types of FAIM2 antibodies detecting N-terminus (FAIM2-abN) and C-terminus (FAIM2-abC) were used for FAIM2 IHC analysis. Antibody specific to synaptophysin, a known NE tumor marker, was used for parallel direct comparison. While SCLC sections with two different FAIM2 antibodies showed moderate to strong FAIM2 expression, no detectable or less intense synaptophysin expression was observed in the adjacent SCLC sections, suggesting that FAIM2 may outperform synaptophysin as a diagnostic marker of SCLC.

**Figure 4:**
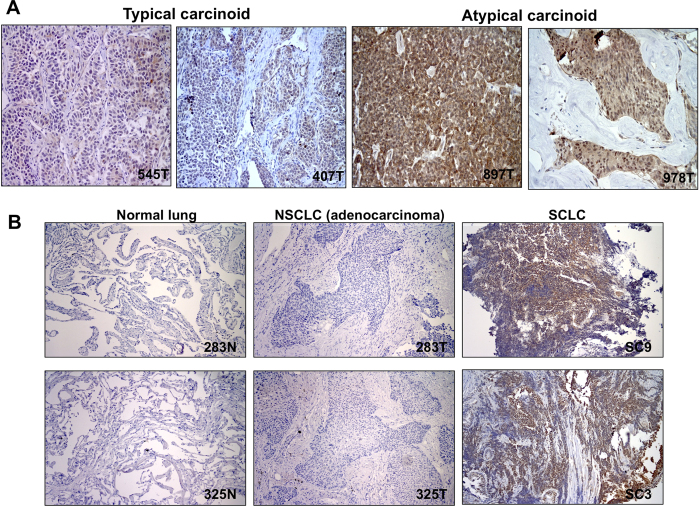
IHC analysis of FAIM2 expression in lung carcinoids, lung adenocarcinomas, and normal lung tissues in comparison to SCLC. (**A**) FAIM2 protein expression was measured using a FAIM2 antibody (FAIM2-abN) in typical carcinoids (545T and 407T) and atypical carcinoids (897T and 978T). While strong FAIM2 expression was detected in atypical carcinoids, little or relatively lower FAIM2 expression was detected in typical carcinoid tumors. (**B**) FAIM2 expression by IHC analysis was compared between SCLCs, lung adenocarcinomas, and normal lung. While strong FAIM2 expression was detected in SCLCs (SC3 and SC9), FAIM2 expression was not detected in normal lung tissues (283N and 325N) or in lung adenocarcinomas (283T and 325T).

To examine FAIM2 as a SCLC diagnostic marker, we performed IHC on biopsy sections from 32 SCLC patients. Out of four FAIM2 antibodies commercially available, two antibodies showing stain-positivity were selected for further evaluation. These two antibodies recognize different residues on FAIM2 protein – one recognizes residues in the N-terminal (FAIM2-abN) and the other recognizes residues close to the center (FAIM2-abC). IHC using synaptophysin, an established NE tumor marker, was performed in parallel to compare to the performance of IHC using FAIM2. FAIM2-abN and –abC both showed positive staining mainly in the nucleus with partial cytoplasmic reactivity. Among 32 SCLCs tested, FAIM2 expression was detected with more than 51–76% tumor cell positivity in 40% (13/32) by FAIM2-abN and 68% (22/32) by FAIM-abC. In comparison, synaptophysin expression was observed in 32% (10/31) of SCLCs tested, and in most tumors was noted to have less intense staining. No detectable synaptophysin staining was observed in 68% (21/31) of SCLCs. Overall, IHC using FAIM2 (P2 = 93.8%) significantly outperformed IHC using synaptophysin (P1 = 31.3%) in SCLCs (*p *< 0.001) (Fig. 3). A table summarizing IHC results of synaptophysin and FAIM2 is shown (Table S1). Table S1 was made using a cut-off level of 51% of expression in tumor cells. To further study FAIM2 expression in other pulmonary NE tumors and lung cancer, we performed IHC on lung carcinoids, lung adenocarcinomas, and normal lung tissue. In lung carcinoids, strong nuclear positivity with partial cytoplasmic staining was observed (Fig. 4A). When the two carcinoid subtypes were directly compared, atypical carcinoids, the more aggressive form of carcinoid, showed higher FAIM2 expression as observed in qRT-PCR. This suggests that FAIM2 may serve as a better diagnostic marker for poor prognostic types of pulmonary NE tumors including atypical lung carcinoids and SCLCs. To investigate if FAIM2 expression is specific to SCLC, we included 8 pairs of NSCLC lung adenocarcinomas and matched normal tissues. Although FAIM2-abC had focal positive reactivity in respiratory epithelium, lymphocytes, and pneumocytes in the normal lung tissues, both FAIM2-abN and FAIM2-abC showed negative staining in lung adenocarcinomas (Fig. 4B). This indicates that the specificity of FAIM2 expression for SCLCs may be a useful adjunct to classic markers and could lead to more accurate diagnosing of subtypes of NE tumors.

### Inhibitory role of FAIM2 in Fas-induced apoptosis and therapeutic advantage of FAIM2 knockdown on drug response

**Figure 5:**
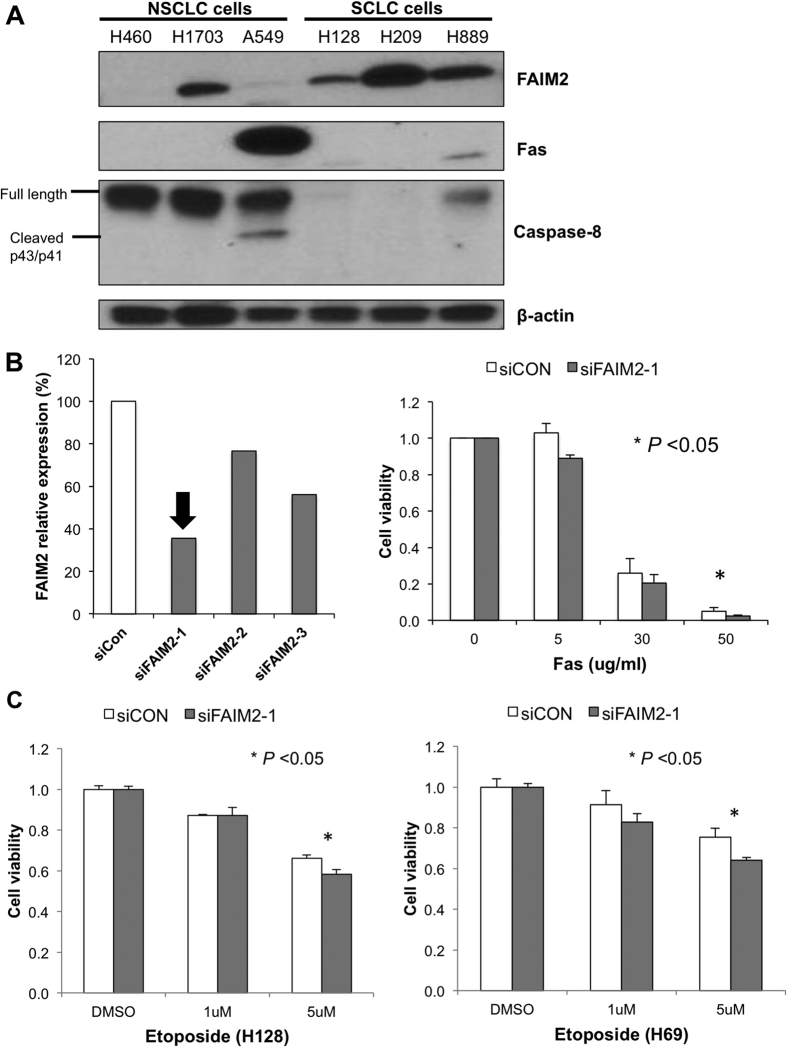
FAIM2 inhibits the Fas-apoptosis pathway and inhibition of FAIM2 expression significantly decreases cell viability in response to Fas and etoposide treatment in SCLC cells. (**A**) Western blot analysis of FAIM2, Fas and caspase-8 in SCLC and NSCLC cells. An inverse correlation between FAIM2 and proapoptotic proteins, Fas and Caspase-8 expression was observed. FAIM2 was significantly expressed in all three SCLC cell lines while only one NSCLC cell line (H1703) showed a clear FAIM2 expression. There was a very clear inverse correlation between FAIM2 and Fas and a modest correlation between FAIM2 and Caspase-8. Beta-actin used as an internal control. (**B**) We tested three siRNAs targeting FAIM2 and selected the most efficient one (siFAIM2-1) in the knockdown of FAIM2 expression (left panel). Cell viability was measured in the presence of Fas agonist to induce apoptosis after the transfection of FAIM2 siRNA. Cell viability was significantly reduced by FAIM2 inhibition (*p* < 0.05) (right panel). (**C**) Etoposide treatment synergistically reduced cell viability in SCLC cells (H128 and H69) with FAIM2 knockdown compared to that in non-silencing control (*p* < 0.05).

To better understand the role of FAIM2 in SCLCs, we obtained SCLC cell lines to interrogate the Fas apoptosis cascade. Three SCLC (H128, H209, and H889) and 3 NSCLC (H460, H1703, and A549) cells were used to explore the cascade by Western blot analysis. All three SCLC cells expressed FAIM2 while only one NSCLC cell showed comparable FAIM2 expression to SCLC cells (Fig. 5A). Interestingly, caspase 8 showed the most differential expression between SCLC and NSCLC cells with inverse correlation to FAIM2 expression. This result is consistent with what we observed with CCLE database (Fig. 1A). As Fas downstream components have only minimal expression in SCLC cells, we suspect that endogenous apoptotic signaling through this cascade might not be active in these cells. Therefore, we treated these cells with a Fas agonist to induce apoptosis and compared responses between SCLC cells with FAIM2 knockdown and control cells (Fig. 5B). H889 cells were treated with siFAIM2 or control siRNA (siCON) and further treated with Fas agonist 24 hr after siRNA transfection. In response to different Fas concentration, cells with FAIM2 knockdown showed decreased cell viability compared with non-silencing control cells (Fig. 5B). This suggests that blocking FAIM2 expression induces more apoptotic cells, and this might be used as a potential therapeutic strategy in treating SCLCs.

Next, to explore the therapeutic effect of FAIM2 modulation, drug sensitivity was measured in combination with FAIM2 knockdown in SCLC cells. Etoposide is an anti-cancer drug currently used in the treatment of SCLC patients. Upon FAIM2 knockdown, cells were treated with etoposide for 72 hours and then cell viability was measured with an MTS assay. In two SCLC cell lines tested (H69 and H128), FAIM2 knockdown improved sensitivity to etoposide treatment (*p *< 0.05) (Fig. 5C). These data suggest that inhibition of FAIM2 can be a potential therapeutic strategy to enhance drug response in SCLCs.

## Discussion

SCLC has a poor prognosis and limited treatment options. Discovering molecular markers specific for NE tumors and SCLC may reveal both diagnostic markers and novel therapeutic targets. By searching the CCLE genomic database we identified apoptosis related genes which were differentially expressed in SCLC compared to NSCLC (Fig. 1A) and focused specifically on the genes involved in Fas-apoptosis pathway. We found in SCLC that while genes promoting apoptosis (*FADD, FAS*, and *CASP8*) were down-regulated, *FAIM2* was significantly overexpressed in the CCLE dataset. Although there are some overlaps of the expression of markers in Fig 1, each one showed a statistically significant difference of expression between NSCLC and SCLC. This suggested that Fas-apoptosis pathway might play a different role for the development of different types of lung cancer. This observation of *FAIM2* overexpression was then confirmed at the level of transcription (Fig. 1B,C) and protein expression (Fig. 5A) in multiple SCLC and NSCLC cell lines. We therefore identified *FAIM2* as a gene of interest as a novel diagnostic marker and a therapeutic target for SCLC and other lung NE tumors.

Typical and atypical carcinoids are low- and intermediate-grade lung NE tumors respectively and show different patterns of tumor growth and clinical prognosis. While examination of mitoses and necrosis is frequently used in classifying these two subtypes, it can be challenging to make a clear distinction between these two similar types of NE tumors. In this study, we observed that *FAIM2* mRNA levels in atypical carcinoids are significantly overexpressed compared to typical carcinoids (*p *= 0.007) (Fig. 2). The number of samples used in this study may not be enough to make a concrete conclusion for the diagnostic use of FAIM2 in small cell lung cancer and atypical carcinoid samples. A further study analyzing a large number of small cell lung cancer and atypical carcinoid samples will be required to prove the clinical usefulness of FAIM2.

In addition, our data strongly supports that IHC using an efficient anti-FAIM2 antibody can be very helpful for diagnosing SCLC, and that FAIM2 may be more specific for SCLC than established NE markers such as synaptophysin^2^. We believe that FAIM2 alone or combined with other existing NE markers can improve diagnostic accuracy in SCLC and other NE tumors but to validate the significance of FAIM2 as a diagnostic marker of SCLC a larger validation study will be necessary.

We then analyzed whether a knock-down of FAIM2 can reduce the tumor growth. FAIM2 inhibits cell deaths by Fas-mediated mechanisms. Thus, we tested whether two apoptotic molecules, Fas and Caspase-8, are inhibited by FAIM2. As expected, Fas and caspase-8 showed almost no or very weak protein expression in SCLC cell lines (Fig. 5A). This suggests that inhibition of anti-apoptosis molecules by FAIM2 may restore repressed apoptosis signaling and eventually lead to tumor cell death. After confirming that FAIM2 has a high protein expression and inhibits expression of apoptosis proteins in SCLC, we transfected SCLC cells with FAIM2 siRNA and measured cell viability. The MTS cell viability assay showed a significant reduction in cell viability after FAIM2 siRNA transfection compared to control (*p *< 0.05) (Fig. 5B).

Etoposide and cisplatin is the standard chemotherapeutic regimen in SCLC so to examine a role for FAIM2 inhibition in SCLC we looked for synergistic effects between FAIM2 inhibitor and etoposide treatment in two SCLC cell lines. As shown in Fig. 5C, a significant reduction of cell viability (*p *< 0.05) was observed when SCLC cells were treated with both etoposide and FAIM2 siRNA. There are few effective chemotherapy agents in SCLC and all tumors quickly develop resistance to treatment. New therapeutic targets are an area of interest and our new candidate gene, *FAIM2*, should undergo further testing as a new targeted therapy candidate in SCLC. FAIM2 inhibition showed modest but significant tumor killing effect (*p* < 0.05). Although this is statistically significant for tumor killing effect, FAIM2 inhibition alone would not be enough for SCLC treatment. However, as there is no effective treatment for SCLC at this time, this new finding might suggest a new option for SCLC treatment, ideally combined with another anti-cancer therapeutic agents.

This is the first study to identify *FAIM2* as a diagnostic marker in SCLC and other NE tumors. We have demonstrated that *FAIM2* can distinguish between atypical and typical carcinoids and between SCLC and NSCLC tumors as a selective and specific diagnostic marker. Knock-down of FAIM2 by siRNA shows that inhibition of anti-apoptotic mechanisms lead to cell death and might be a new therapeutic option to SCLC. We observed additive effects with FAIM2 inhibition and etoposide *in vitro* suggesting a possible role in combined treatment approaches. FAIM2 represents an exciting new target in SCLC and further studies are warranted to validate the potential of FAIM2 as a diagnostic marker and a therapeutic target in SCLC and other NE tumors.

## Methods

### Samples

Sixteen typical and eight atypical carcinoid and matched normal tissue samples from University of California, San Francisco (UCSF) were used for *FAIM2* Taqman expression analysis and FAIM2 protein immunohistochemical staining. Eight pairs of normal lung and matched lung adenocarcinoma samples from UCSF were also used for FAIM2 protein staining. All these samples were collected under the IRB protocol (#11-06107) approved by the Committee for Human Research at the University of California, San Francisco. Additional 32 SCLC samples collected under the approved IRB by Kosin University College of Medicine, Republic of Korea were used for FAIM2 protein staining. All experiments were carried out in accordance with the approved guidelines.

### *FAIM2 mRNA expression analysis*

Twelve lung cancer cell lines (H1299, H358, H889, H1666, H2087, H69, H128, H209, H460, SNU-1327, SNU-1330, and SNU-2292) were obtained from either ATCC (www.atcc.org) or Korean Cell Line Bank (http://cellbank.snu.ac.kr). These 12 lung cancer cell lines were used for real-time quantitative PCR analysis. Briefly, RNA from these cell lines was extracted using RNeasy Mini Kit (Qiagen, Cat. #: 74104). Extracted RNAs (500 ng) were used for cDNA synthesis using SuperScript^®^ III First-Strand Synthesis System (ThermoFisher Scientific, Cat. #: 18080-051). Ten ng of Synthesized cDNAs were analyzed in triplicate using two *FAIM2* Taqman probes (ThermoFisher Scientific, Hs00392342_m1 (X2-3) and Hs00392345_m1 (X5-6)) and *ACTB* endogenous control probe in ABI 7900HT system (ThermoFisher Scientific). The normalized *FAIM2* transcript level was determined by calculating dCt values by subtracting mean Ct values of *FAIM2* with mean Ct values of ACTB. Then, 1/dCt values were calculated to obtain representative values indicating higher values corresponding to higher transcript levels. For semi-quantitative RT-PCR, primers detecting *FAIM2* transcript were designed by Primer 3 and cDNAs were amplified by 30 cycles according to standard PCR amplification.

### Immunohistochemistry

Sections (5 μm in thickness) were deparaffinized and hydrated in xylene and varying concentrations of ethanol, and then steamed in 10 mM sodium citrate solution from Biogenex for 20 minutes for antigen retrieval. Endogenous peroxidase was quenched with a 3% H_2_O_2_ solution (Sigma) for 10 min at room temperature. Blocking was performed with 5% goat serum (Zymed) diluted in TBS containing 0.1% Tween-20 for one hour at room temperature before overnight incubation with the primary antibodies at 4 °C. After washing three times in TBS containing 0.1% Tween-20 for 5 min, sections were incubated with a biotinylated secondary antibody anti-rabbit (Vector Laboratories) or anti-mouse (Vector Laboratories) at a dilution of 1:500 for 1 hour at room temperature. Sections were washed with TBS-T, incubated in ABC reagent (Vectastain ABC kit, Vector) according to the manufacturer’s instructions, and developed using DAB (Sigma). Primary antibodies were used as follows: anti-FAIM2 (abN, Novus); anti-FAIM2 (abC, ABgent); anti-synaptophysin (Milipore). Negative control sections were prepared by the exclusion of the primary antibody to determine the specificity of the staining. Tissue sections were then examined microscopically on an Olympus photomicroscope (Inha, Japan).

The immunohistochemical staining was evaluated by the intensity and the proportion of positive tumor cells. The cell-positive score was graded: <5%, 0 (−); 5–25%, 1 (+); 25–50%, 2 (++); 51–76%, 3 (+++); and >76%, 4 (++++). In order to evaluate the intensity, tumor cells were compared with non-neoplastic epithelium. Cytoplasmic and nuclear staining in tumor cells, whose staining property was stronger than that in non-neoplastic epithelium, was considered positive. The staining intensity score was graded: 0, negative (−); 1, weak (+); 2, moderate (++); and 3, strong staining (+++).

### Western blot analysis for Fas apoptosis pathway

Whole cell lysates were prepared from cell lines (H460, H1703, A549, H128, H209, and H889) with RIPA lysis buffer. Western blot analysis was performed using 50 μg of protein extracts per lane, electrophoresed, transferred to PVDF membranes (Millipore), and immunoblotted with anti-FAIM2 (Novus), anti-Fas (Milipore), caspase 8 (Cell Signaling Technology), and anti-β-actin (AC–15, Sigma). The membranes were washed and treated with rat anti-species IgG secondary antibody conjugated to horseradish peroxidase (Amersham Pharmacia). The antigen -antibody reactions were visualized by using enhanced chemiluminescence assay ECL (Amersham Pharmacia) and exposed to enhanced chemiluminescence film.

### siRNA-mediated FAIM2 knockdown, Fas-induced apoptosis, and etoposide treatment

Cell culture was done with RPMI medium 1640 (Gibco) containing 10% FBS (Gibco), 100 U/ml of penicillin, 100 mg/ml of streptomycin, and 2 mM L-glutamine. siRNAs for FAIM2 (FlexiTube GeneSolution) and non-silencing control were obtained from Qiagen. 2 × 10^6^ cells were seeded in 6-well plates the day before transfection. siRNAs were transfected into the cells using lipofectamine 2000 (Invitrogen) according to the manufacturer’s instructions. For analyzing FAIM2 knockdown, cells were harvested 48 hours after transfection and RNAs were extracted as previously described. For Fas-induced apoptosis assay, cells transfected with siRNAs were re-plated into 48-well plate in 2% serum conditioned RPMI media and treated with ant-Fas (Milipore) at various concentrations in triplicates. After 24 hours incubation, cell viability was determined by MTS assay using CellTiter-Glo assay (Promega).

### Cell viability (MTS) assay

Cell viability was measured using Promega CellTiter-Glo^®^ Luminescent Cell Viability Assay kit and Synergy HT Multi-Mode Microplate Reader (Biotek).

## Additional Information

**How to cite this article**: Kang, H. C. *et al*. FAIM2, as a novel diagnostic maker and a potential therapeutic target for small-cell lung cancer and atypical carcinoid. *Sci. Rep.* **6**, 34022; doi: 10.1038/srep34022 (2016).

## Supplementary Material

Supplementary Information
