# Supplementary material for: FAIM2, as a novel diagnostic maker and a potential therapeutic target for small-cell lung cancer and atypical carcinoid
Source: Sci Rep. 2016 Sep 28;6:34022. doi: 10.1038/srep34022 (PMC5039724; doi:10.1038/srep34022)
Supplement: Supplementary Information [file srep34022-s1.docx]

**Supplementary Information**

**FAIM2, as a novel diagnostic maker and a potential therapeutic target for small-cell lung cancer and atypical carcinoid**

Hio Chung Kang^1,2^, Jong In Kim^1,3^, Hee Kyung Chang^4^, Gavitt Woodard^1^, Young Sik Choi^5^, Ja-Lok Ku^6^, David M. Jablons^1,2^, and Il-Jin Kim^1,2^

^1^Thoracic Oncology Laboratory, Department of Surgery, ^2^Comprehensive Cancer Center, University of California San Francisco, San Francisco, CA, USA,

^3^Department of Thoracic and Cardiovascular Surgery, ^4^Department of Pathology, ^5^Department of Internal Medicine, Kosin University College of Medicine, Busan, Republic of Korea, ^6^Laboratory of Cell Biology, Cancer Research Institute, Seoul National University College of Medicine, Seoul, Republic of Korea.

Correspondence and requests for materials should be addressed to I.J.K (email: [Il-Jin.Kim@ucsf.edu](mailto:Il-Jin.Kim@ucsf.edu)) or D.M.J (email: [David.Jablons@ucsf.edu](mailto:David.Jablons@ucsf.edu))

Keywords: FAIM2, pulmonary neuroendocrine tumor, small-cell lung cancer (SCLC)

**Supplementary Figure**

**
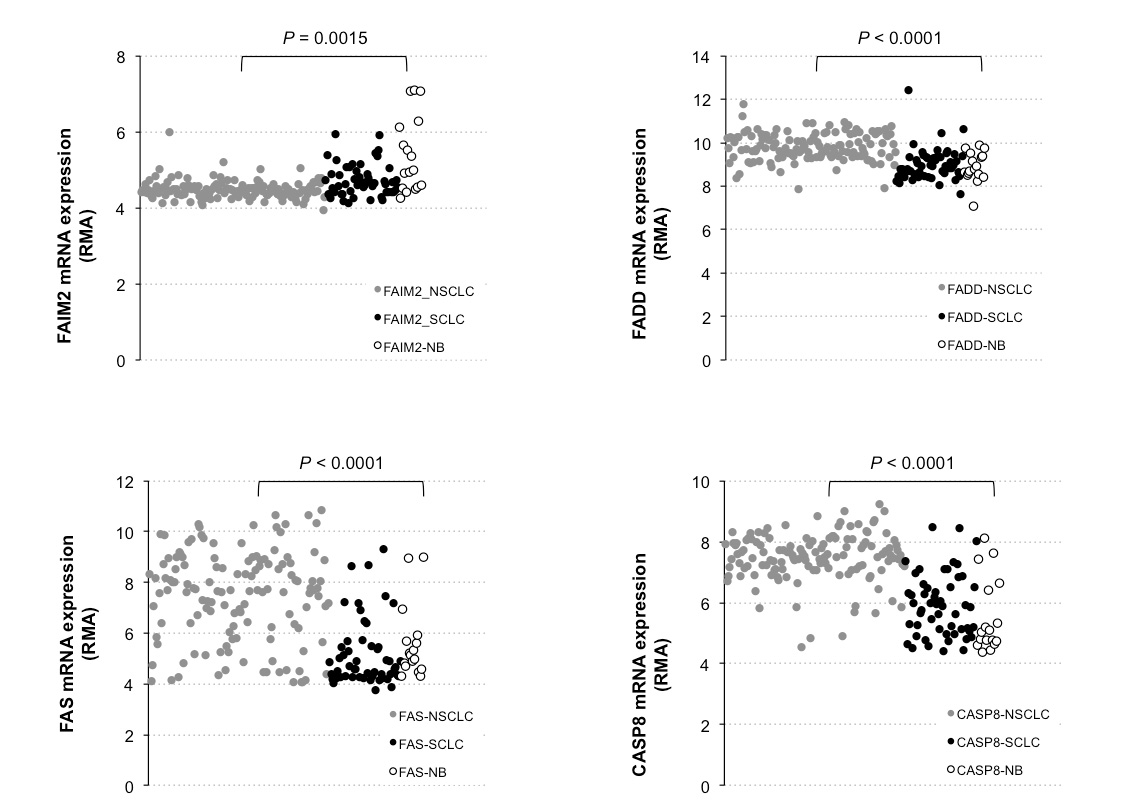
**

**Figure S1. Expression analysis of *FAIM2, FADD, FAS*, and *CASP8* in NSCLC, SCLC, and neuroblastoma (NB) cell lines.** 133 NSCLC, 54 SCLC, and 17 neuroblastoma (NB) cell lines from CCLE database (<http://www.broadinstitute.org/ccle>) were analyzed. *FAIM2* mRNA expression was significantly higher in NB cells compared to those in NSCLC cells (*p* = 0.0015). In contrast, three proapoptotic genes, *FAS, FADD*, and *CASP8* showed higher expression in NSCLC compared to those NB cells (*p* < 0.0001 for *FADD, FAS*, and *CASP8*). These data suggest tumor cells (SCLC and NB) with neuroendocrine origin commonly have a suppressed Fas apoptosis signaling pathway and overexpression of the anti-apoptotic gene *FAIM2*.

**Supplementary Table.**

**Table S1. Summary of IHC results of synaptophysin and FAIM2 in SCLC**

|  | **No. of SCLCs < 51% IHC positivity** | **No. of SCLCs >= 51% IHC positivity** | **P value (P1 vs P2)** |
| --- | --- | --- | --- |
| **synaptophysin (P1)** | 32 (100%) | 0 (0%) | ***p* < 0.001** |
| **FAIM2-abN (P2)** | 19 (60%) | 13 (40%) |  |
| **FAIM2-abC (P2)** | 10 (31%) | 22 (69%) |  |
